# Simultaneous Trapping of Two Types of Particles with Focused Elegant Third-Order Hermite–Gaussian Beams

**DOI:** 10.3390/mi12070769

**Published:** 2021-06-29

**Authors:** Jingjing Su, Nan Li, Jiapeng Mou, Yishi Liu, Xingfan Chen, Huizhu Hu

**Affiliations:** 1 State Key Laboratory of Modern Optical Instrumentation, College of Optical Science and Engineering, Zhejiang University, Hangzhou 310027, China; sujj@zju.edu.cn (J.S.); 21730074@zju.edu.cn (J.M.); 21930047@zju.edu.cn (Y.L.); mycotty@zju.edu.cn (X.C.); 2 Quantum Sensing Center, Zhejiang Lab, Hangzhou 310000, China

**Keywords:** optical trapping, third-order Hermite–Gaussian beam, radiation force, Rayleigh scattering theory

## Abstract

The focusing properties of elegant third-order Hermite–Gaussian beams (TH_3_GBs) and the radiation forces exerted on dielectric spherical particles produced by such beams in the Rayleigh scattering regime have been theoretically studied. Numerical results indicate that the elegant TH_3_GBs can be used to simultaneously trap and manipulate nanosized dielectric spheres with refractive indexes lower than the surrounding medium at the focus and those with refractive indexes larger than the surrounding medium in the focal vicinity. Furthermore, by changing the radius of the beam waist, the transverse trapping range and stiffness at the focal plane can be changed.

## 1. Introduction

Optical trapping and manipulation of particles have demonstrated significant progress in recent applications in the fields of micromachines, biology, and colloidal chemistry [1,2,3]. Previously, the conventional optical tweezers or optical trap was constructed with a highly focused Gaussian beam, and it was used to capture particles with high refractive indexes, larger than that of the surrounding medium in the focal region [4,5]. Recent theoretical studies on radiation forces demonstrated that a beam with a Gaussian-like intensity profile should be used to trap a refractive index greater than that of the ambient medium. A beam with a hollow-like intensity profile is applicable in capturing a refractive index lower than that of the ambient medium. In comparison with the bright spot in the conventional high refractive index particle trap, the realization of a low refractive index particle trap needs zero central intensity, which inevitably requires complex beam-shaping technology. Various methods have been used to generate hollow-like intensity profile beams: the hollow optical fibers [6], geometrical optical [7], transverse mode selection [8], and computer-generated hologram methods [9]. Several types of hollow-like intensity profile beams have been constructed in recent years, with Laguerre–Gaussian [10,11], circular airy [12,13,14], higher-order Bessel [9,15], multi-Gaussian Schell-model [16], and hollow Gaussian beams [17,18,19] being the most common types of beams. At present, holographic beam-shaping or interference pattern-realizing dark space beam has also been used to capture low-refractive-index particles [20]. To the best of our knowledge, the focusing properties of hollow elegant third-order Hermite–Gaussian beams (TH_3_GBs) have not been studied.

The Hermite–Gaussian beams are extensively used in the fields of electron acceleration, nonlinear optics, free-space optical communication, and optical manipulation [21,22,23,24,25]. To date, the trapping characteristics of different Hermite–Gaussian beams, such as Hermite–Gaussian correlated Schell-model [26], Hermite–Gaussian vortex [21], and partially coherent Hermite–Gaussian array beams have been studied [27]. Since Siegman introduced new Hermite–Gaussian solutions known as elegant Hermite–Gaussian modes that satisfy the paraxial wave function, [28] studies on the focusing properties of the elegant Hermite–Gaussian beam have garnered increasing attention. Zhao studied the trapping characteristics of elegant Hermite-cosine-Gaussian beams [29], whereas Luo studied the radiation forces of elegant Hermite-cosh-Gaussian beams [30]. Although these two types of beams produce a dark hollow beam profile at the focal plane and simultaneously trap particles of high and low refractive indexes, both beams are modulated by sinusoidal factors. We found that the simplest form of the elegant third-order Hermite–Gaussian beam composed of the third-order Hermitian-polynomial and Gaussian functions can also simultaneously capture two kinds of particles with different refractive indexes in the optical trap.

The optical force that allows trapping and manipulation of particles are produced by the transfer of angular momentum and momentum from the electromagnetic field to the particles. Particles change the momentum and angular momentum flux of the beam by scattering. Therefore, the calculation of light force is essentially the calculation of light scattering [31,32,33]. In this paper, we have derived the analytical expression of the elegant TH_3_GBs exerted on the high and low refractive index particles in the Rayleigh scattering regime. The hollow elegant third-order Hermite–Gaussian beam is also a hollow beam after focusing, and there is a dark region in the center of the focal plane along with a doughnut configuration in the focal vicinity; thus, the low-refractive-index particles can be captured at the focus. Moreover, the electromagnetic energy at the center of the hollow beam is very low, and the scattering force acting on the particle trapped at the focal point is very small; therefore, the particles are not easily damaged owing to a reduction of heat absorption. Finally, we analyze the stable capture conditions for the effective capture and manipulation of particles.

## 2. Materials and Methods

In our discussion, the electric field distribution of the doughnut elegant TH_3_GBs at $z_{1}=$0 is expressed as follows:

(1) $$E_{1}(r_{1},z_{1}=0)=A_{0}H_{3}(\frac{r_{1}}{w_{0}})\exp(-\frac{r_{1}{}^{2}}{w_{0}{}^{2}})$$

(2) $$A_{0}=\sqrt{\frac{P}{6\pi n_{m}\varepsilon_{0}cw_{0}{}^{2}}}$$

(3) $$H_{3}(\frac{r_{1}}{w_{0}})=8{\left(\frac{r_{1}}{w_{0}}\right)}^{3}-12\frac{r_{1}}{w_{0}}$$

where *A_0_* is determined by the incident power *P*. Term $w_{0}\;$denotes the waist radius of the input Gaussian beams, whereas $n_{m}\;$denotes the refractive index of the surrounding medium (liquid). Terms $r_{1}=\sqrt{x_{1}^{2}+y_{1}^{2}}$ and z_1_ indicate the transverse and axial coordinates, respectively, in the input plane of the incident beam. Term *H*_3_ represents the third-order Hermite polynomials.

**Figure 1:**
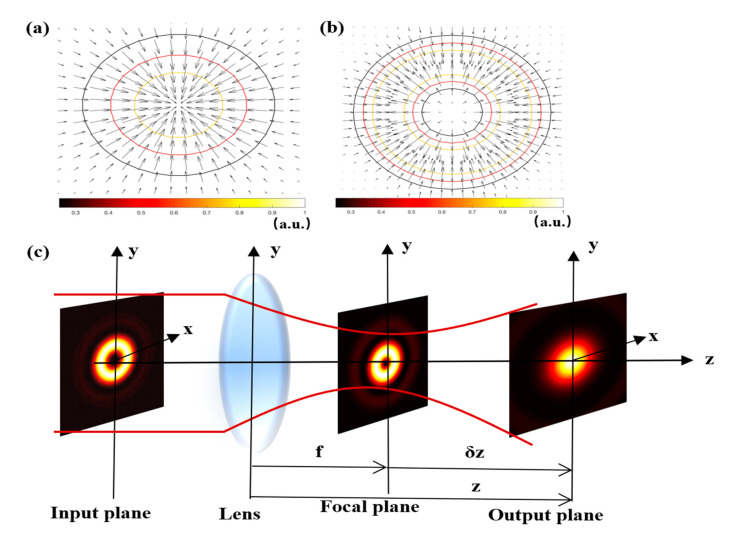
Spatial distribution of the fundamental Gaussian beams (**a**) and the elegant TH_3_GBs (**b**) at the input plane. (**c**) shows the schematic of the elegant TH_3_GBs. The intensity distribution of the elegant TH_3_GB is represented at different positions along the z-axis (at the input plane, at the focal plane, and the output plane located at *δz* = 2 µm after the focal plane. where z is the longitudinal coordinate at the beginning of the focusing lens, z=f+δz, δz is the distance from the focal point on the axis and f is the focal length of the thin lens. The colors represent the normalized magnitudes of the radiation forces. The directions and lengths of the black arrows represent the directions and magnitudes of the resultant forces.

It is well known that when the refractive index of the particle is larger than that of the surrounding medium, the gradient force directs the particles to the region of maximum intensity. When the refractive index of the particle is smaller than that of the surrounding medium, the gradient force has the opposite direction and guides the particle to the region of smaller light intensity. From Figure 1a, we observe that the arrows representing the electromagnetic field intensity gradient of the focused Gaussian beam are directed towards the centers, and the directions and lengths of the arrows represent the directions and magnitudes of the resultant forces. Gaussian beams are usually used to trap high-index (with respect to the surrounding medium) particles. In comparison with the fundamental Gaussian beams, the gradient force distribution of the elegant TH_3_GBs is almost absent at the center, and it appears as a ring distribution, as indicated in Figure 1b. In the field of optical tweezing, it has been revealed that the focused dark hollow trap has some advantages over the conventional optical tweezers for minimizing photodamage on the trapped particles in experimental trapping. At the same time, the gradient force characteristics of low refractive index particles show that the center of the dark hollow trap can be used to capture low refractive index particles. Compared with the fundamental Gaussian beams, the elegant TH_3_GBs have a doughnut-shaped intensity distribution at the input plane, so the performance of the optical tweezers would be improved.

Now, we consider the elegant TH_3_GBs propagation through a thin lens focusing system, as shown in Figure 1c. The focal length of the thin lens is located at the input plane with *f* = 5 mm, and *z* is the axial distance from the input plane to the output planes.  λ=1064 nm is the wavelength of the input wave in the medium. A, B, C, and D are the transfer matrix elements of the lens optical system.

(ABCD)=(1z01)(10−1f1)=(1−zfz−1f1)

Under the framework of paraxial approximation, the propagation of light beams through an optical ABCD system are determined by the extended Huygens–Fresnel diffraction integral [34]. Using the integral formula Equation (4) and substituting Equations (1)–(4) into Equation (5), the propagation formula of the TH_3_GBs at the cylindrical coordinates are derived and obtained as follows:

(4) $$\int_{0}^{\infty }r^{u}\exp(-a_{1}r^{2})J_{0}(pr)\mathrm{dr}=\Gamma (\frac{1+u}{2})\frac{1}{2a_{1}{}^{(u+1)/2}}F_{1}(\frac{1+u}{2},1,-\frac{p^{2}}{4a_{1}})$$

(5) $$\begin{array}{cc} E(r,z) & =\frac{i2\pi A_{0}}{\lambda B}\exp(\frac{ikD}{2B}r^{2})\\ \; & \int_{0}^{\infty }H_{3}(\frac{r_{1}}{w_{0}})\exp(-\frac{r_{1}{}^{2}}{w_{0}{}^{2}})J_{0}(\frac{krr_{1}}{B})\exp(-\frac{ikA}{2B}r_{1}{}^{2})r_{1}dr_{1}\\ \; & =\frac{iA_{0}}{\lambda B}\exp(ikz)\exp(\frac{ikD}{2B}r^{2})\\ \; & \left\{\frac{16\pi }{w_{0}{}^{3}}\Gamma (\frac{5}{2})\frac{1}{2a_{1}{}^{5/2}}F_{1}(\frac{5}{2},1,-\frac{p^{2}}{4a_{1}})-\frac{24\pi }{w_{0}}\Gamma (\frac{3}{2})\frac{1}{2a_{1}{}^{3/2}}F_{1}(\frac{3}{2},1,-\frac{p^{2}}{4a_{1}})\right\} \end{array}$$

where $a_{1}=\frac{1}{\omega_{0}^{2}}+i\frac{kA}{2B}$ and $p=k\frac{r}{B}$. $r_{1}$ and $r=\sqrt{x^{2}+y^{2}}$ denote radial coordinates in the input and output planes, respectively. _1_*F*_1_ is the Kummer confluent hypergeometric function. Term $k=2\pi /\lambda =k_{0}n_{m}$ represents the wavenumber with $n_{m}$.$\;\;k_{0}$ denoting the wave number in a vacuum, whereas $n_{m}$ denotes the refractive index of the surrounding medium (liquid).

**Figure 2:**
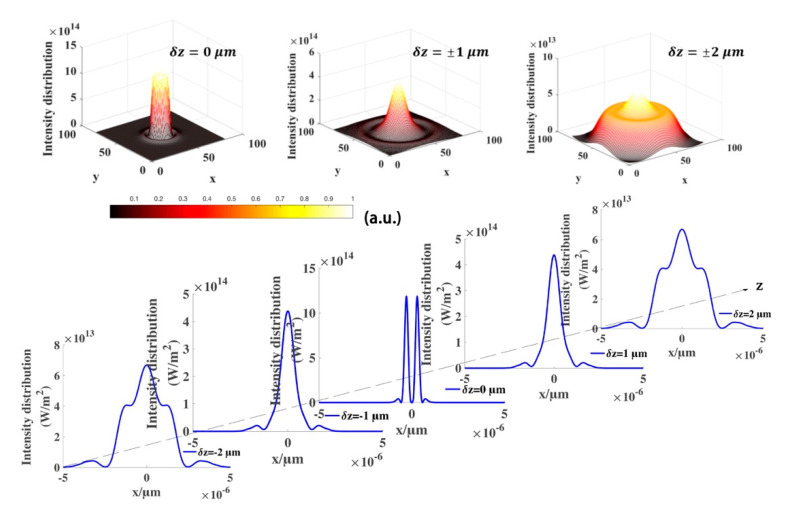
Evolution of the intensity distribution of the focused TH_3_GBs from δz=−2 μm to δz=2 μm around z=5 mm. In these simulations, we select the beam power P=1 W,$\;\omega_{0}=5\;\mathrm{mm}$, and λ=1064 nm.

The evolutions of the focusing characteristics of the elegant TH_3_GBs versus x for several δz are illustrated in Figure 2. Term δz represents the distance between the focal and output planes. It is clearly observed from Figure 2 that the intensity distribution is sensitive to δz. TH_3_GBs has rotational symmetry of the doughnut-shaped intensity at δz=0 μm. We find that the intensity of the beams is doughnut-shaped at the center of the focusing plane and tiny side lobes are located near the main peaks, therefore, low refractive index particles can be trapped at the dark center of the focal plane of the focused beam. Away from the focus (by decreasing or increasing *δz*), the intensity profiles of the focused beam gradually transform into a single peak distribution with a maximum intensity at its center. The hollow profile of the elegant TH_3_GBs disappears. Owing to the focus prosperities of the elegant TH_3_GBs, we expect these beams to be used for capturing two kinds of particles with different refractive indexes.

## 3. Results

### Radiation Forces Produced by the Focused Elegant TH_3_GBs

The radius of particles is assumed to be sufficiently smaller than the wavelengths of laser beams. The Rayleigh dielectric particles can be treated as a simple point dipole in the light fields. The radiation force can be calculated using the following expressions [32,35]:

(6) FGrad=14ε0εmRe(β)∇|E2|

(7) $$F_{scat}=\frac{\varepsilon_{0}\varepsilon_{m}{}^{3}k_{0}{}^{4}}{12\pi }\left|\beta^{2}\right|\left|E^{2}\right|$$

(8) $$\beta =4\pi a^{3}\frac{\varepsilon_{p}-\varepsilon_{m}}{\varepsilon_{p}+2\varepsilon_{m}}$$

where *β* is the polarizability of the Rayleigh particle, $\varepsilon_{m}=n_{m}^{2}$ and $\varepsilon_{p}=n_{p}^{2}$ denote the dielectric function of the Rayleigh particle and that of the surrounding medium, respectively. Term *a* is the radius of the particle. $k_{0}$ denotes the vacuum wave number and $\varepsilon_{0}$ is the dielectric constant in a vacuum. The refractive index of the ambient is $n_{m}=1.33$ (i.e., water), whereas that of the high-refractive-index particle and low-index particle is $n_{p}=1.592$ (i.e., polystyrene) and $n_{p}=1$ (i.e., air bubble), respectively. In the subsequent calculations, we consider a particle of radius a=20 nm.

**Figure 3:**
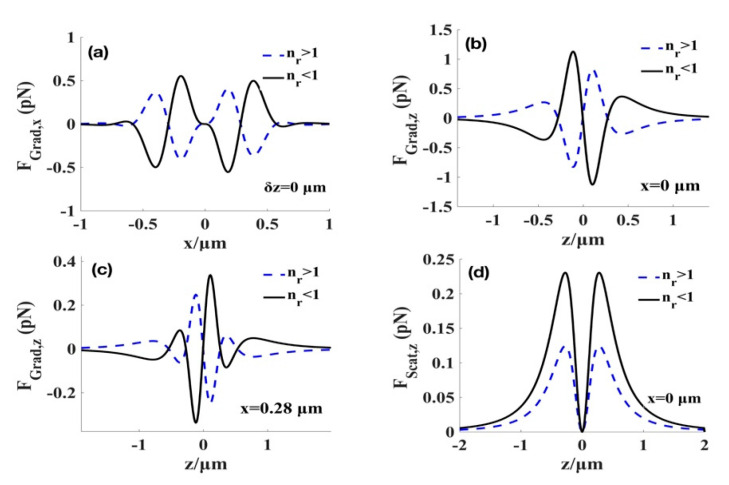
Radiation forces produced by the elegant TH_3_GBs on high- (blue dashed curve) and low-index particles (black solid curve). (**a**) Transverse gradient force at the focal plane. (**b**) Longitudinal gradient force at the focal point; (**c**) Longitudinal gradient force at the point x=0.28 μm. (**d**) Scattering force at the focal point. We select a sphere with a radius a=20 nm and $n_{r}=n_{p}/n_{m}$ represents the relative refractive index. $n_{m}=1.332$ is the refractive index of the surrounding field, and the high and low refractive indices are the homogeneous Rayleigh particles. Other parameters are λ=1.064 μm, $w_{0}=5\;\mathrm{mm}$, f=5 mm, P=1 W.

Figure 3 illustrates the distributions of the longitudinal and transverse radiation forces of the focused elegant TH_3_GBs exerted on the high-index ($n_{p}=1.592)$ and low-index ($n_{p}=1$) particles. The sign of the gradient force represents the direction of the force: for the positive $F_{Grad,x}$ the transverse gradient force is along the +x direction, whereas for the negative $F_{Grad,-x}\;$is along the -x direction. Similarly, for positive (negative) $F_{Grad,+z}$, the longitudinal gradient force is in the +z (−z) direction. The scattering force is always along the +z direction (as can be seen in Figure 3b). From Figure 3a,b, we can observe that there is an equilibrium point at the focus for the low-index particles, and the gradient force along the z-direction (as can be seen in Figure 3b) is always larger than the forward-scattering force as shown in Figure 3d. This indicates that the particles with a low index can be stably trapped by the elegant TH_3_GBs at the focus. From Figure 3d, we note that the scattering acting on the low-index particle at the focus of the focused elegant TH_3_GBs force is zero. From Figure 3a,c, we find that two equilibrium points are present (x=±0.28 μm) near the focus where the high-index particle ($n_{p}=1.592)$can be trapped. Therefore, Figure 3 demonstrated that the focused elegant TH_3_GBs can simultaneously manipulate or trap two types of particles, and this is superior to the fundamental Gaussian beams that have no equilibrium point for low-index particles at the focus.

**Figure 4:**
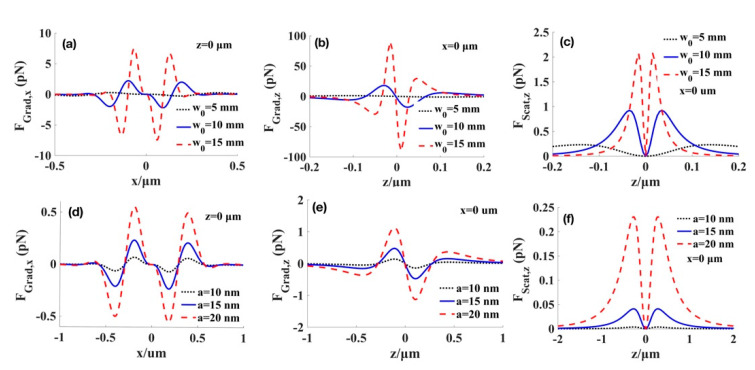
Effect of waist radius of the beams (**a**–**c**) at particles’ radius a=20 nm, and radius of particles (**d**–**f**) at waist radius of the beams $w_{0}=5\;\mathrm{mm}$ on the radiation force for the low-index particles with ($n_{p}=1$). (**a**,**d**) transverse gradient force at the focal plane. (**b**,**e**) longitudinal gradient force at the focal point. (**c**,**f**) scattering force at the focal point.

The effects of the waist radius of the beams and those of particles through the radiation forces exerted on the low-index particles are indicated in Figure 4. From Figure 4a–c, we find that as the waist radius of the beam increases, both the gradient and scattering forces increase, but the transverse region of trapping particles shrinks. Therefore, the larger value of *ω*_0_ corresponds to the easier trapping for the low-index particles. Similarly, by increasing the radius of particles in Figure 4d–f, the radiation forces also increase, but the transverse trapping range is not affected by the radius of particles. Consequently, the stiffness of the optical trap can be enhanced by adjusting the value of *ω*_0_.

**Figure 5:**
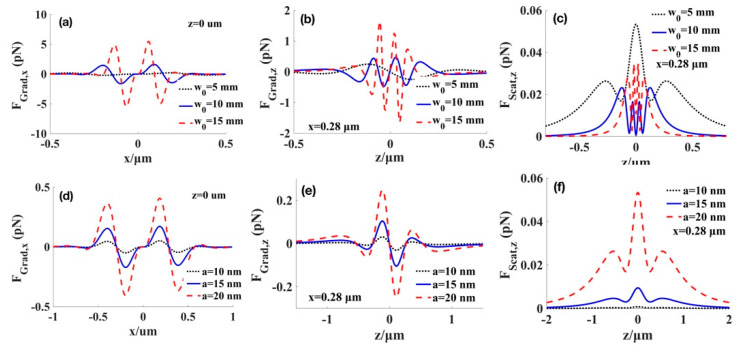
Effect of waist radius of the beams (**a**–**c**) at particles’ radius a=20 nm, and radius of particles (**d**–**f**) at waist radius of the beams $w_{0}=5\;\mathrm{mm}$ for the high-index particles with ($n_{p}=1.592$). (**a**,**d**) transverse gradient force at the focal plane. (**b**,**e**) longitudinal gradient force at the point x=0.28 μm. (**c**,**f**) scattering force at the point x=0.28 μm.

Figure 5 illustrates the changes of the gradient and scattering forces exerted on the high-index particles for several values of the waist radius of the beams and those of the particles. The transverse gradient forces increase as the value of the waist radius increases, similar to the case of low-index particles as shown in Figure 5a. Figure 5b–c depicts the longitudinal gradient force at the point x=0.28 μm, whereas Figure 5e,f plots the scattering force at the point x=0.28 μm. Figure 5b,c show that the position of the trapped high-index particles is closely related to the value of waist radius. From Figure 5d–f, it can be found that when the radius of particles becomes larger, the radiation force will also become larger; thus, the magnitudes of transverse and longitudinal gradient forces can be modulated by the radius of particles without affecting the trapping range. Figure 5c,f show that compared with the longitudinal gradient force in Figure 5b,e, the magnitude of the scattering forces is significantly smaller than the axial gradient force.

## 4. Discussion

Based on the above analysis, there are still several necessary conditions for stably trapping particles using the elegant TH_3_GBs. The first necessary criterion for axial stability is that the backward longitudinal gradient force should be sufficiently greater to overcome the forward scattering force, which is shown in Figure 3b,d for low-index particles. Similarly, Figure 3c and Figure 5c show the longitudinal gradient force and scattering force at x=0.28 μm, respectively, for high-index particles. Therefore, the first stability criterion is well-fulfilled. Second, because the particle is significantly small (a≪λ), it suffers from the Brownian motion owing to the thermal fluctuation from the ambient (e.g., water). For stable trapping, the potential well of the gradient force trap must be larger to conquer the Brownian force. This condition can be determined using the fluctuation-dissipation theorem of Einstein, the magnitude of the Brownian force can be calculated by $F_{B}=\sqrt{\;12\pi \eta ak_{B}T}\Gamma \left(t\right)$ where $\eta =7.977\times 10^{-4}\;\mathrm{Pa}\cdot s$ is the viscosity of water at room temperature, T=300 K, $k_{B}$ is the Boltzmann constant, *Γ*(*t*) is a normalized Gaussian white-noise process and a=20 nm [36,37,38]. Adopting the above parameters, we obtain the value of the Brownian force,$\;F_{B}$, which is approximately $1.6\times 10^{-3}\;\mathrm{pN}$, we established that the gradient force exerted on the two types of particles are larger than the Brownian force from Figure 3a–c. Therefore, the magnitude of the Brownian force of the Rayleigh particles is much smaller than the gradient forces, and they could be ignored.

## 5. Conclusions

In this study, we present the analytical expression for the propagation of the elegant TH_3_GBs using a paraxial ABCD optical system. Based on the extended Huygens-Fresnel principle and Rayleigh scattering regime, we investigated the focusing properties of the elegant TH_3_GBs. Owing to the dark hollow beam profile produced at the focal plane, the energy of the elegant TH_3_GBs at the focus is very low; thus, the heat absorbed by the particles could be significantly reduced to avoid damage to the particle. Subsequently, we show that this beam can simultaneously capture high refractive index spheres on the focal plane. In addition, it is demonstrated that the trapping stiffness and transverse trapping range increase as the value of the waist radius increases. Finally, we explicitly analyze the trapping stability. Our results have theoretical reference values in the field of optical micromanipulation and optical tweezers.

## Data Availability

Not applicable.
